# Bioactive Hydrogels: Design and Characterization of Cellulose-Derived Injectable Composites

**DOI:** 10.3390/ma14164511

**Published:** 2021-08-11

**Authors:** Andrea Fiorati, Cristina Linciano, Camilla Galante, Maria Grazia Raucci, Lina Altomare

**Affiliations:** 1 Department of Chemistry, Materials, and Chemical Engineering “G. Natta”—Politecnico di Milano, Piazza Leonardo da Vinci 32, I-20133 Milano, Italy; cristina.linciano@mail.polimi.it (C.L.); camilla.galante@mail.polimi.it (C.G.); lina.altomare@polimi.it (L.A.); 2 INSTM National Interuniversity Consortium of Materials Science and Technology, Politecnico di Milano Local Unit, Piazza Leonardo da Vinci 32, I-20133 Milano, Italy; 3 Institute of Polymers, Composites and Biomaterials (IPCB), National Research Council (CNR), Viale J.F. Kennedy, 54 Mostra d’Oltremare Pad. 20, 80125 Naples, Italy; mariagrazia.raucci@cnr.it

**Keywords:** TEMPO-oxidized nanocellulose, hydrogel, biocomposite, mineralization, hydroxylapatite

## Abstract

Cellulose represents a low cost, abundant, and renewable polysaccharide with great versatility; it has a hierarchical structure composed of nanofibers with high aspect ratio (3–4 nm wide, hundreds of μm long). TEMPO-mediated oxidation represents one of the most diffused methods to obtain cellulose nanofibers (CNFs): It is possible to obtain physically crosslinked hydrogels by means of divalent cation addition. The presence of inorganic components, such as calcium phosphates (CaP), can improve not only their mechanical properties but also the bioactivity of the gels. The aim of this work is to design and characterize a TEMPO-oxidized cellulose nanofibers (TOCNFs) injectable hydrogel embedded with inorganic particles, CaP and CaP-GO, for bone tissue regeneration. Inorganic particles act as physical crosslinkers, as proven by rheological characterization, which reported an increase in mechanical properties. The average load value registered in injection tests was in the range of 1.5–4.4 N, far below 30 N, considered a reasonable injection force upper limit. Samples were stable for up to 28 days and both CaP and CaP-GO accelerate mineralization as suggested by SEM and XRD analysis. No cytotoxic effects were shown on SAOS-2 cells cultured with eluates. This work demonstrated that the physicochemical properties of TOCNFs-based dispersions could be enhanced and modulated through the addition of the inorganic phases, maintaining the injectability and bioactivity of the hydrogels.

## 1. Introduction

Hydrogels are macromolecular polymeric materials that are crosslinked to form a 3-D network. They are able to retain relatively large volumes of water, without solubilization in an aqueous environment and have assumed increasing interest as scaffolds for tissue regeneration support due to their tunable properties. Moreover they are able to mimic the extracellular matrix environment, and, when combined with specific compounds that enhance their mechanical properties, might give rise to a biocomposite able to cover many applications in the biomedical field [1,2,3,4].

Natural polymer-based hydrogels, properly crosslinked through chemical or physical bonds, allow us to obtain a biocompatible and three-dimensional structure whose mechanical properties and degradation kinetics can be modulated in different ways and targeted to a specific application [5]. Among natural polymers, cellulose represents a low cost, abundant and renewable polysaccharide with great versatility [6,7,8].

Generally, cellulose chains are assembled into hierarchical fibrillary architectures which can be disassembled into nanometric dimensioned fibers [6,9]. Among all the different methods to obtain cellulose nanofibers (CNFs) proposed in the literature, the selective oxidation of C6 cellulose hydroxyls catalyzed by the stable radical 2,2,6,6-Tetramethyl-1-piperidinyloxy (TEMPO) represents one of the most diffused approaches [10,11,12,13,14]. From TEMPO-oxidized CNFs (TOCNFs) aqueous dispersions it is possible to obtain physically crosslinked hydrogels by means of divalent cation addition [15,16,17,18]. Recently, cellulose and its derivative have been widely investigated leading to different applications in the biomedical field [19]. Even though cellulose by itself does not present specific adhesion sites, this material can be easily modified by blending, functionalization or by incorporating inorganic particles. For all these reasons, cellulose-based biomaterials offer some important advantages and lead to different applications in the biomedical field, such as tissue regeneration and drug delivery, and have been widely studied, demonstrating cellulose biocompatibility, cytocompatibility and mechanical stability [15,20].

The main disadvantage of the physical crosslinked hydrogels is their lack of mechanical properties [10]. The realization of biocomposite hydrogels through inorganic particles incorporation can help in overcoming this drawback. Hydroxyapatite and its precursors (CaP), and graphene-oxide (GO) have been widely investigated as bioactive fillers for polymer matrices. HA has attracted increasing attention as a substitute material for bone thanks to its crystallographic similarity to calcified human tissues; it can be retrieved from natural sources or be synthesized with high variability in purity, ratio of amorphous to crystalline phase, particles size and distribution [21]. Moreover, its bioactivity makes it a suitable material to realize biocomposites that enhance mineralization, cell attachment and migration [22].

The high functionalization of the GO surface enhances its biocompatibility and facilitates its incorporation in polymer matrix [23]. This structure provides binding sites that improve cell adhesion and promote stem cell differentiation processes, particularly adipogenesis and osteogenesis. GO is proposed as an ideal reinforcing material that can be used to strengthen and toughen HA without compromising its bioactivity and biocompatibility thanks to its high chemical inertness [24]. GO has also shown antibacterial features and has been tested as an effective treatment for cancer as it can specifically target cancer cells with a significant cytotoxic effect against osteosarcoma [25].

Depending on the specific application, cellulose-based hydrogel can be combined with different composites enhancing its specific properties. In particular, it has been demonstrated that the incorporation of inorganic particles, such as HA and GO, can improve its mechanical and bioactive properties [24].

**Figure 1:**
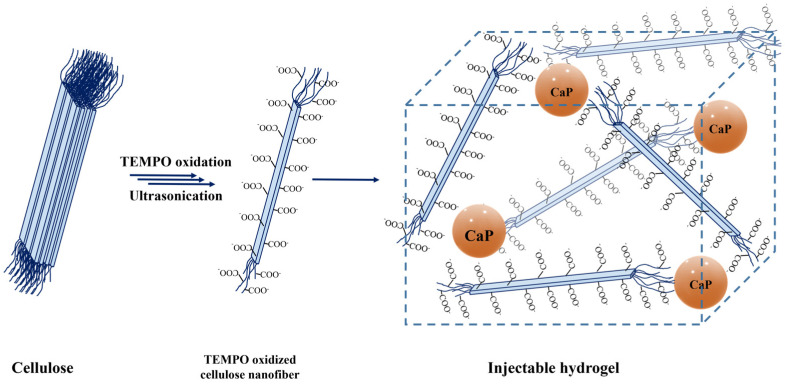
Schematic representation of the production of TOCNFs-CaP/CaPGO hydrogels. The pristine cellulose was converted to TEMPO oxidized cellulose nanofibers by means of TEMPO/KBr/NaClO oxidative system followed by an ultrasonic assisted dispersion. Inorganic particles were added to the achieved dispersion in order to induce the sol-gel transition.

In this work, TEMPO-oxidized cellulose nanofibers (TOCNFs) hydrogels embedded with inorganic particles were prepared, as shown in Figure 1. Their formulation was chosen in order to achieve an injectable and bioactive matrix for bone tissue regeneration. In particular, the injectability was studied firstly by means of a rheological characterization, and then exploiting an ad hoc developed setup able to measure the force needed to extrude the biocomposites loaded in a syringe equipped with an 18 G needle. After we verified that the obtained hydrogels were suitable for our purpose, in vitro assays were performed in order to investigate their behavior in terms of swelling, ability to mineralize, and release of cytotoxic components.

## 2. Materials and Methods

All chemicals were commercially available and were provided by Merck Life Science S.r.l. (Milan, Italy). For this study Sigmacell (Lot No. SLBZ6032) was chosen as cotton linter cellulose standard. Sodium hypochlorite solution Lot No. K52670914 031.

### 2.1. Composite Hydrogels Preparation

#### 2.1.1. Oxidized Cellulose Synthesis

Oxidized cellulose was obtained as described in the literature [10,12]. Briefly, 0.538 g of TEMPO (3.44 mmol) and 3.70 g of KBr (31.1 mmol) were dissolved in deionized (DI) water (1 L) under magnetic agitation. Then this solution was added to a dispersion of cellulose (25 g in 375 mL of water) under vigorous stirring. Sodium hypochlorite solution (125 mL, 6–14% active chlorine) was gradually added in 1 h under stirring. During the reaction, the pH was continuously monitored and maintained in the range of 10.5–11.0 through the progressive addition of NaOH solution (2 M, about 100 mL). After keeping it under constant stirring for 16 h, HCl solution (2 M) was added until pH 2 was reached. The oxidized cellulose pulp was collected by filtration on a Büchner and extensively washed with HClaq (0.01 M, 3 × 0.5 L), water (3 × 0.5 L) and ethanol (4 × 0.5 L). The obtained oxidized cellulose was then dried in an oven at 37 °C for 24 h.

The number of acidic moieties introduced was evaluated by means of colorimetric titration employing NaOH_aq_ (0.1 M) and phenolphthalein as indicator. The oxidation degree was 2.13 mmol_COOH_/g_cellulose_.

#### 2.1.2. CaP and CaPGO Particles Synthesis

CaP particles, with and without GO, were produced at room temperature by sol-gel technology as previously reported [24]. In brief, calcium nitrate tetrahydrate [Ca(NO_3_)_2_·4H_2_O] and di-ammonium phosphate [(NH_4_)_2_HPO_4_], at Ca/P molar ratio in the range of 1.60–1.70 were used as precursors of Ca^2+^ and PO_4_^3−^ respectively. The preparation of CaPGO particles was obtained by adding GO (Nanesa srl, Arezzo, Italy, 1.5 wt. %) during CaP in situ sol-gel synthesis. In particular, GO exfoliation was performed in distilled water (dH_2_O) by sonication in ultrasound bath for several hours. Then, GO solution (1.5 wt. %) was added in Ca^2+^ solution (3 M) in order to improve the interactions between calcium ions and chemical groups on the GO surface. After 30 min of magnetic mixing at room temperature, PO_4_^3−^ solution (3.58 M) was gradually added and the pH was set to pH 10–11 by NH_4_OH solution. After gelification, the system was washed several times to remove some residues of reaction, such as ammonium nitrate (NH_4_NO_3_), and then was freeze-dried for 2 days to obtain the powders.

#### 2.1.3. Composite Hydrogels Synthesis

A TOCNFs stock dispersion (4% w w^−1^) was prepared as follows: 2.0 g of oxidized cellulose were weighed in a beaker and dispersed in DI water (about 30 mL), then 1 equivalent of NaOH_aq_ (2 M) with respect to the content of carboxylic groups was added. Then, the slurry was sonicated using a sonic dismembrator (FB-505, Fisherbrand, 6 mm probe tip working in alternated mode (40 s on, 10 s off), with an output power of 40% the nominal value (200 W), until the dispersion appears clear and transparent (approximately 40 min). The overheating of the dispersion was prevented by means of an ice bath. When the dispersion was achieved, the pH was set to 7.4 by means of NaOH_aq_ (0.1 M), the weight was adjusted to 50 g with DI water and then was autoclaved at 121 °C for 20 min.

The specimens were prepared by mixing the stock solution and the inorganic particles dispersed in DI water, as summarized in Table 1. The inclusion process was conducted in a sterile environment by loading the two components into two different sterile syringes connected by means of a three-way Luer Stopcock and then mixing them with 50 passes between syringes until the particles homogeneously spread in the suspension. 

### 2.2. Characterizations

#### 2.2.1. FTIR and XRD Analyses

Fourier transform infrared spectroscopy (FT-IR) was conducted using infrared-grade KBr disks and a Varian 640-IR spectrometer (Agilent Technologies, Santa Clara, CA, USA) in the 400–4000 cm^−1^ range (4 cm^−1^ resolution, 32 scans).

X-ray diffraction (XRD) experiments were performed with Panalytical Empyrean diffractometer (Malvern Panalytical Ltd., Malvern, UK) equipped with a Bragg Brentano geometry (Cu-Kα1 radiation; λ = 0.154056 nm). The X-ray diffraction patterns were collected in 0–80 2θ range (scan step size = 0.02°, scanning time as per step = 20 s, room temperature). Each measurement was performed in triplicate in order to increase the signal-to-noise ratio. XRD patterns were analyzed using Profex Software [26].

#### 2.2.2. Thermal Analyses

Thermogravimetric analysis (TGA) and differential Thermal Analysis (DTA) were carried on with Perkin Elmer STA 6000 (Perkin Elmer Italia S.p.A, Milan, Italy) on freeze-dried TOCNFs hydrogels samples employing a heating ramp of 10 °C min^−1^ in the range of 30–900 °C, under air conditions.

#### 2.2.3. Morphological Characterization

The morphology and microstructure of freeze-dried TOCNFs hydrogels pre- and post-mineralization assays were observed under a Zeiss EVO 50 EP (Carl Zeiss Microscopy Gmb, Jena, Germany) scanning electron microscope (SEM) equipped with a Bruker Quantax 200 6/30 detector for Energy-dispersive X-ray spectroscopy (EDS) analysis. ETH was set at 20.0 kV, I probe ranged from 50 to 100 pA and specimens were observed without gold sputter coating, in low vacuum conditions using the back-scattered (BDS) and Variable Pressure Secondary Electron (VPSE) detectors. SEM images were analyzed using ImageJ Software [27].

#### 2.2.4. Rheological Characterization

Rheological tests were performed on TOCNFs hydrogels in duplicate using an Anton Paar MCR 302 Modular Compact rheometer (Anton Paar GmbH, Graz, Austria).

The tests were conducted using a parallel plates geometry (diameter D = 25 mm) while the temperature was kept constant at 20 °C. Prior to each measurement, preconditioning was performed at a constant shear strain of 10% and at molar frequency of 100 Hz for 1 min followed by a 2 min rest [28,29].

The rheological properties of the hydrogels were evaluated by means three different experiments: (i) Strain sweep, increasing the shear strain from 0.01 to 100% with constant frequency at 1 Hz in order to determinate the linear viscoelastic range (LVR) of the material (Figure S1). The strain value (10%) was selected in order to optimize the signal-to-noise ratio for the following experiments; (ii) frequency sweep, increasing the frequency from 0.01 to 100 Hz with 10 % of strain; (iii) shear rate sweep, increasing the shear rate from 0.1 to 1000 s^−1^.

#### 2.2.5. Injectability Test

Injectability tests were performed following a custom experimental setup (Figure S2) coupled to a uniaxial tensile testing machine (MTS 1/MH, load cell 5 kN, MTS Systems, Eden Prairie, MN, USA) in compression mode. The setup consists of a Poly(methyl methacrylate) cylinder perforated in order to accommodate the syringe allowing the maintenance of the vertical position and the alignment to the testing machine plate. Underneath the syringe needle, a container is positioned to collect the extruded sample.

Hydrogels were loaded inside a new, sterile 5 mL syringe and extruded through an 18 G needle. The syringe was mounted on the predefined setup; the plate was first gradually approached to the syringe piston at a speed of 1 mm min^−1^ until a load of 0.15 N was detected indicating successful contact between the plate and piston. After that, the speed was set to 0.6 mm s^−1^ (corresponding to flow rates of approximately 2.28 mL min^−1^) for 15 mm and the force applied was recorded [30]. At the end of extrusion, the plate was lifted, and the procedure was repeated in duplicate on the same syringe for each TOCNF type extruding another 15 mm of sample.

The experiment was carried out both on samples immediately after loading in the syringe and on samples that rested inside the syringe for 48 h in order to evaluate the differences in terms of viscosity and thixotropic characteristics.

Injectability measurements were performed at room temperature (20 °C).

### 2.3. In Vitro Test

#### 2.3.1. Swelling Test

The swelling tests were conducted by dipping the samples (500 mg) in sterile Dulbecco’s Modified Eagle Medium (DMEM, 4 mL) enriched with antibiotic (penicillin and streptomycin 1 % w w^−1^) and 0.02% (w w^−1^) sodium azide (NaN_3_), in order to prevent microbiological contamination and incubated them at 37 °C. The culture medium was removed, and the samples were weighed at progressive time steps, starting from a 15-min interval for a total of 34 days. Then the hydrogels were covered with 4 mL of fresh sterile culture medium and stored again in the incubator.

The percentage change in weight (*W*_%_) was calculated using Equation (1):

(1) $$W_{\%}=\frac{\left(W_{f}-W_{i}\right)}{W_{i}}\times 100$$

where, *W_i_* is the initial weight of the sample before soaking it in the culture medium, while *W_f_* is the weight of the sample following the removal of the medium.

#### 2.3.2. In Vitro Mineralization Test

The apatite formation and bioactivity of the samples were evaluated by simulated body fluid (SBF) treatment. 1.5 × SBF was prepared according to the method proposed by Kokubo et al. [31]. To prepare 1 L of SBF solution, 0.5 L of water was put under continuous stirring in a plastic beaker and heated to 36 ± 0.5 °C. Then, the following reagents were sequentially dissolved into the solution: NaCl (8.035 g, 137.5 mmol), NaHCO_3_ (0.355 g, 4.2 mmol), KCl (0.225 g, 3.0 mmol), K_2_HPO_4_·3H_2_O (0.231 g, 1.0 mmol), MgCl_2_·H_2_O (0.311 g, 1.5 mmol), 1 M HCl (39 mL), CaCl_2_ (0.292 g, 1.9 mmol), Na_2_SO_4_ (0.072 g, 0.5 mmol). When the solution again reached 36 ± 0.5 °C, Tris (hydroxyl methyl) methyl amine (6.118 g, 50.5 mmol) was gradually added. Finally, the pH was set to 7.4 by addition of HCl_aq_ (1 M) then water was added to have 1 L of SBF solution, which was stored at 4 °C.

For mineralization tests, samples were accurately weighed (600 mg), placed inside a strainer, and immersed in 1.5 × SBF (20 mL). Samples were placed in a Shaker Incubator (SKI 4, Argo Lab, Giorgio Bormac S.r.l Carpi MO, Italy) at 50 rpm, 37 °C and SBF was renewed every 48 h. The mineralization process was conveyed at four steps: 7, 14, 21 and 28 days. In particular, 21–28 days hydrogels were washed 3 times in water for 10 min in order to remove NaCl interferences.

#### 2.3.3. Cytotoxicity Assay

Samples were prepared according to the procedure described in par 2.1.3, both TOCNFs and inorganic particles were dispersed in DMEM instead of water.

In order to evaluate the effect of possible release of cytotoxic compounds from hydrogels, in vitro indirect cytotoxicity test was carried out according to EN ISO 10993-12 using SAOS-2 human osteosarcoma cell line. Cells were seeded in 96-well tissue culture polystyrene (TCPS) plates (1×10^4^ cells per well) and cultured in DMEM (supplemented by 1 mM sodium pyruvate, 10% fetal bovine serum, 4 mM L-glutamine, and 1% penicillin-streptomycin) at 37 °C in presence of 5% CO_2_ humidified atmosphere, until 70% confluence was reached.

Culture medium eluates were obtained by dipping 200 µL of each sample in 1 mL of medium (time points 1, 3 and 7 days, each time point was repeated four times). Cells were then cultured for 24 h with culture medium eluates or fresh culture medium. Cell viability was assessed by alamar Blue assay; fluorescence was read by a spectrophotometer (Synergy H1 spectrophotometer, BioTek, Rodano, Italy; λ_exc_ = 540 nm, λ_em_ = 595 nm). The percentage cell viability was calculated according to equation 2, where *f* is the fluorescence value of eluates, control and Alamar Blue reference.

(2) $$Cell\;viability\left[\%\right]=\frac{f_{eluates}-f_{AlamarBlue}}{f_{control}-f_{AlamarBlue}}\;\times \;100$$

## 3. Results and Discussion

### 3.1. Composite Hydrogels Preparation and Characterization

#### 3.1.1. FT-IR, XRD and Thermogravimetric Analyses

As reported in the literature [14], the TEMPO-oxidation process leads to the formation of carboxyl groups on the cellulose structure which can be easily recognized by observing the presence of the peak at 1731 cm^−1^, associated with the C=O stretching vibration of the carboxylic acids, in the FT-IR spectrum (Figure S3). These moieties, once properly deprotonated, were exploited to achieve the homogeneous dispersions of TEMPO oxidized cellulose nanofibers. Lastly, the carboxylates were employed for the binding with the CaP and CaPGO particles, acting as cross-linker agent. CaP and CaPGO particles, characterized by means of FT-IR spectroscopy and X-Ray Diffraction (Figure S4), were found to be composed of a mixture of brushite, monetite, hydroxyapatite and other calcium phosphates (e.g., calcium pyrophosphate). Despite the greyish appearance of the CaPGO powders (Figure S4a), the typical FT-IR and XRD signals of graphene oxide cannot be clearly detected due to the low mass ratio and the overlapping with the phosphate groups (Figure S4b,d), as described in the literature [32].

Thermogravimetric analysis performed on freeze-dried samples (Figure S5) shows in all cases a first small weight loss at about 100 °C (about 10%), which was due to the evaporation of adsorbed water. In the range between 200 °C and 300 °C, another relevant weight loss was observed in all samples, associated to the decomposition of the organic content. The combustion of the organic carbon residues was observed at 700 °C, 600 °C, and 400 °C respectively for TOCNFs, TOCNFs CaP and TOCNFs CaPGO samples. As expected, the residue at 900 °C for TOCNFs samples was 0%, while 13.7% and 13.2% of residues were obtained for TOCNFs CaP and TOCNFs CaPGO.

#### 3.1.2. Rheological Characterization

**Figure 2:**
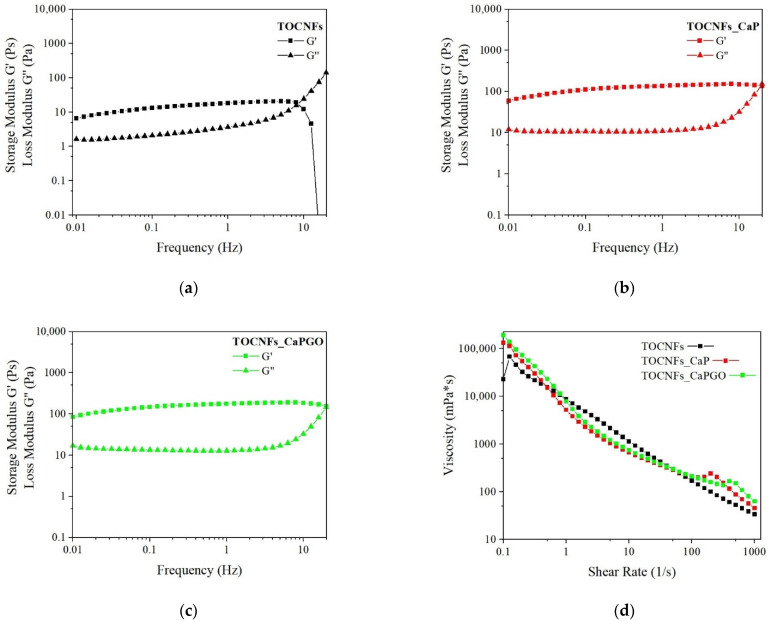
Viscoelastic properties of TOCNFs (black markers), TOCNFs_CaP (red markers) and TOCNFs_CaPGO (Green markers) samples. (**a**–**c**) Dynamic frequency sweeps (20 °C) of composites (10% strain rate); (**d**) Dynamic shear rate sweep (0.1 to 1000 s^−1^).

The dynamic rheological properties of the hydrogels were investigated at 20 °C and reported in Figure 2 and Figure S1. Storage modulus (G’) values remain constant and dominant over loss modulus (G”) values up to about 10 Hz for all the samples. At a specific frequency value, the G’ and G’’ curves intersect, showing a more liquid-like behavior of the hydrogels.

The frequency sweep on TOCNFs samples (Figure 2a) highlights a slight increase in the moduli as the frequency increases, as usually reported for weak physically cross-linked gels [33]. In TOCNFs CaP and TOCNFs CaPGO plots (Figure 2b,c) the G’ and G” curves exhibit a plateau behavior until the crossover point. Their intersect values are higher compared to TOCNFs (Figure 2a), and an increment in the moduli values is observed. G’ values increase from 12 ± 7 Pa in TOCNFs to about 150 ± 40 Pa in TOCNFs Ca and TOCNFs CaPGO. This increase of one order of magnitude suggests an increased gel strength when loaded with HA precursors. The curves remain constant until 30 Hz for TOCNFs CaP and TOCNFs CaPGO and until 7 Hz for TOCNFs samples. These results confirm the capability of the employed HA precursors to act as physical crosslinker by means of the interaction between the particles surface and the cellulose fibers. However, compared to the moduli values reported in the literature for cation-induced gelation of TOCNFs the achieved G’ and G” values are more than one order of magnitude lower [18], even if in this work the concentration of cellulose nanofibers is higher.

Comparing these results with other cellulose-derived hydrogels loaded with calcium phosphate, TOCNFs CaP and TOCNFs CaPGO show a broader LVR. In contrast, the storage modulus values recorded are lower compared with the average G’ values found in these studies [34,35,36]. This is probably a consequence of the higher inorganic content considered. However, considering the valuable results achieved from injectability and mineralization tests, we decided to not explore higher inorganic content.

The viscosity of the three suspensions was measured for shear rate ranging from 0.1 to 1000 s^−1^ (Figure 2d). All the prepared blends exhibit a thixotropic and shear-thinning behavior in this range: the viscosity decreases significantly as the shear rate increases. This represents the typical behavior of non-Newtonian fluids. This phenomenon can be related to the weakening and disruption of the hydrogel network and the consequent release of the entrapped liquid [37]. The shear thinning behavior represents an important characteristic for injectable hydrogels.

#### 3.1.3. Injectability Test

**Figure 3:**
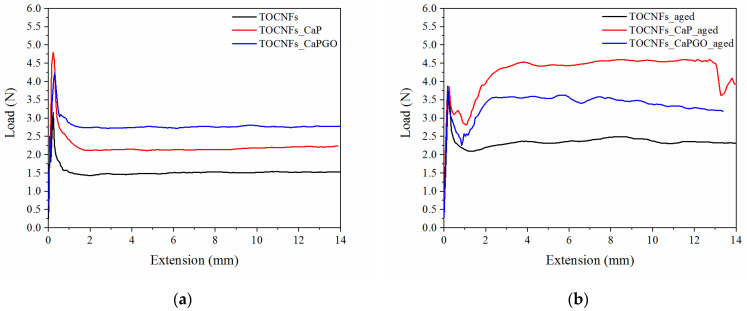
Injectability test: comparison between: (**a**) fresh prepared samples and (**b**) 48 h aged samples. TOCNFs (black line), TOCNFs_CaP (red line) and TOCNFs_CaPGO (blue line) were extruded through a 18 G needle at 20 °C at a crosshead speed of 0.6 mm s^−1^ (2.28 mL min^−1^) measuring the force needed for the process.

The assays were performed by loading the composite hydrogels in a sterile syringe, and then measuring the force needed to extrude the specimens through an 18G needle, at 20 °C at a crosshead speed of 0.6 mm s^−1^ (2.28 mL min^−1^), in order to mimic the operational condition during surgery [30,38]. The reasonable injection force upper limit in bone site is generally around 30 N [30,38,39]. In addition, it is reported that the force required to inject into human tissue is 1.1 times higher than that measured in air [39]. The measured load needed to extrude freshly prepared specimens is reported in Figure 3. All the samples show a force plateau (F_mean_) after an initial force peak (F_max_), which was associated with the force required to begin plunger movement. As expected, TOCNFs samples were more prone to be extruded (F_max_ = 4.52 ± 0.28 N; F_mean_ = 1.51 ± 0.01 N), compared to TOCNFs_CaP (F_max_ = 4.81 ± 0.98 N; F_mean_ = 2.18 ± 0.15 N) and TOCNFs_CaPGO (F_max_ = 4.47 ± 1.02 N; F_mean_ = 2.76 ± 0.03 N). These data are consistent with rheological assays, which have verified that the addition of HA precursors causes an increase in the elastic component of the hydrogels. Moreover, the loading of CaP and CaPGO into the TOCNFs dispersions do not cause obstruction in the needle. In order to evaluate the influence of the thixotropic behavior of the composite hydrogels on injectability, the same measurements were repeated after letting the specimens set for 48 h in the syringes (Figure 3b). As expected, in all cases an increase of the measured forces was recorded (TOCNFs F_max_ = 4.02 ± 0.62 N, F_mean_ = 2.34 ± 0.12 N; TOCNFs CaP F_max_ = 4.99 ± 0.03 N, F_mean_ = 4.37 ± 0.03 N and TOCNFs CaPGO F_max_ = 3.91 ± 0.15 N, F_mean_ = 3.42 ± 0.40 N). All the recorded loads resulted to be far below the 30 N limit, indicating that the proposed composites can be easily injected in vivo at a reasonable rate. Similar injectable hydrogels reported in the literature often require higher load values in order to be successfully extruded and/or lower extrusion speeds [30,39]. As an example, Safwat et al. recently reported TOCNFs hydrogels loaded with a high amount of calcium phosphates which can be extruded trough a 18 G needle at 1.50 N; however, the extrusion speed was set to 1 mm min^−1^, which is 36 times lower than the extrusion rate employed in this work, limiting the clinical applicability of the proposed composite [36].

### 3.2. In Vitro Test

#### 3.2.1. Swelling Test

**Figure 4:**
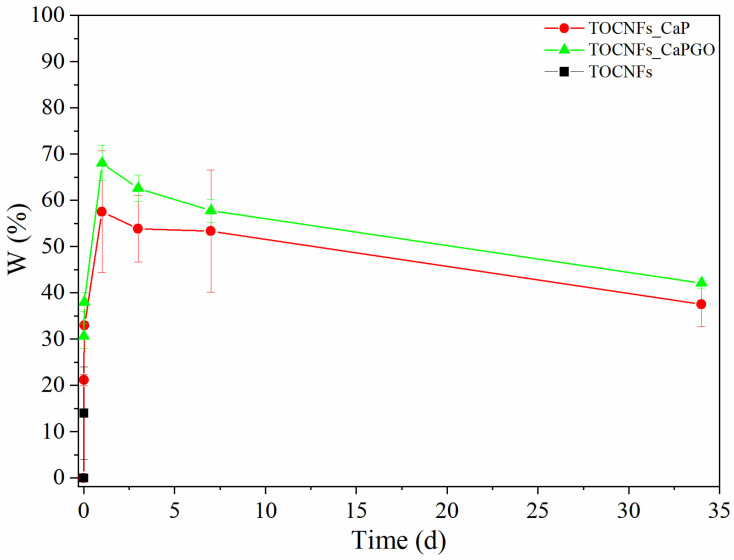
Swelling Test on TOCNFs (black), TOCNFs CaP (red) and TOCNFs CaPGO (green) was performed by immersing the samples in sterile culture medium at 37 °C and carefully weigh the samples at different time steps.

Swelling tests were carried out in order to determine the weight variation and the stability of the biocomposites. Samples were immersed in sterile culture medium (DMEM, enriched with antibiotics and sodium azide) and then incubated at 37 °C. At progressive intervals of 15 min, 1 h and then 1, 3, 7, and 34 days, the culture medium was removed, and the samples were weighed. The results of the swelling test are reported in Figure 4. In case of unloaded TOCNFs dispersions, the samples were totally dissolved after the first time point, while for particles embedded samples, a rapid initial increase was observed within the first 15 min (0.01 days). After 1 day, all samples registered the maximum increase in weight reaching values of 58% for TOCNFs CaP, and 68% for TOCNFs CaPGO. Between 1 and 3 days, a weight loss is observed denoting a possible release of the water used in the gel realization process which is gradually replaced by the culture medium. After 7 days and until the end of the experiment, a further loss in weight was recorded, probably due to a gradual loss and breakage of material during the replacement phase of the culture medium. Nevertheless, the results suggest a good stability of the material.

#### 3.2.2. In Vitro Mineralization Test

Mineralization assays were carried out by aging the hydrogels in simulated body fluid 1.5× (SBF) at 37 °C up to 28 days, and the growth of hydroxyapatite crystals was evaluated as a marker of efficient mineralization. At each time point (7, 14, 21 and 28 days) the samples were freeze-dried and then analyzed by means of XRD and SEM microscopy. The effect of mineralization can be observed also from a macroscopic point of view; indeed, the hydrogels become more opaque and stiffer after a few days.

**Figure 5:**
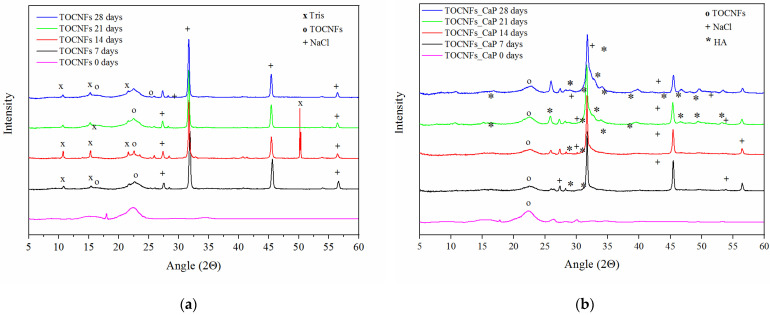

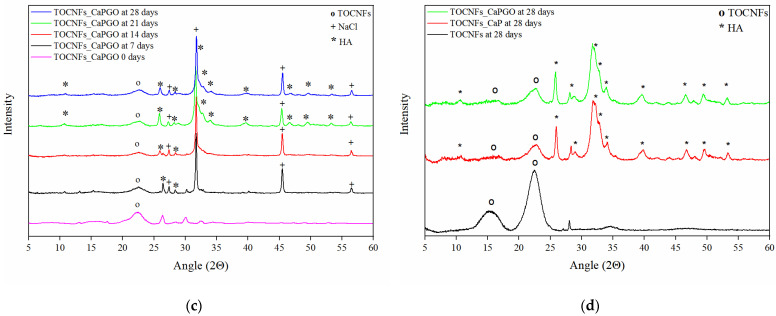
XRD diffraction patterns of nanocomposites hydrogels after 7, 14, 21 and 28 days of different specimens TOCNFs (**a**), TOCNFs CaP (**b**), TOCNFs CaPGO (**c**), and XRD of mineralized hydrogels at 28 days after water rinsing (**d**).

XRD patterns for each sample at all the time points are reported in Figure 5a–c. In all cases, the XRD diffractograms are dominated by the presence of NaCl and Tris(hydroxymethyl) methylamine (Tris), pattern. NaCl was responsible for peaks at 27.5°, 31.7°, 45.6° and 56.6°, while Tris signals were present at 10.6°, 15.3°, 21.6°, 23.5° and 50.3°. The typical peaks of crystalline cellulose Iβ were found at 2θ of 22.5°, 16.5° and 14.7° [40].

In case of TOCNFs samples (Figure 5a) no evidence of apatite formation can be observed. However, in the diffractograms of TOCNFs CaP (Figure 5b) and TOCNFs CaPGO (Figure 5c) samples it was possible to identify the typical diffraction pattern of hydroxyapatite (Ca_10_(PO_4_)_6_(OH)_2_, HA) at 10.6°, 25.9°, 28.3°, 31.8°, 32.8°, 34.0°, 39.6°, 46.7°, 49.5° and 53.2° [32]. In these cases, the presence of HA can be observed even after the first time point (7 days). Interestingly, the HA peaks at 2θ 31.8°, 32.2°, 32.9° and 34.0° appear as a single, broad and poorly defined peak suggesting the nanometric dimensions of the hydroxyapatite crystallite [32]. In order to remove the interference of NaCl and Tris and to verify that HA crystals are tightly embedded in the TOCNFs matrix, after 28 days the hydrogels were rinsed in pure water and the XRD measurements were repeated (Figure 5d). After this washing, the HA crystalline phase becomes more evident confirming that the TOCNFs CaP and TOCNFs CaPGO were successfully mineralized, while hydrogels made of pure TOCNFs do not seem to mineralize at all.

**Figure 6:**
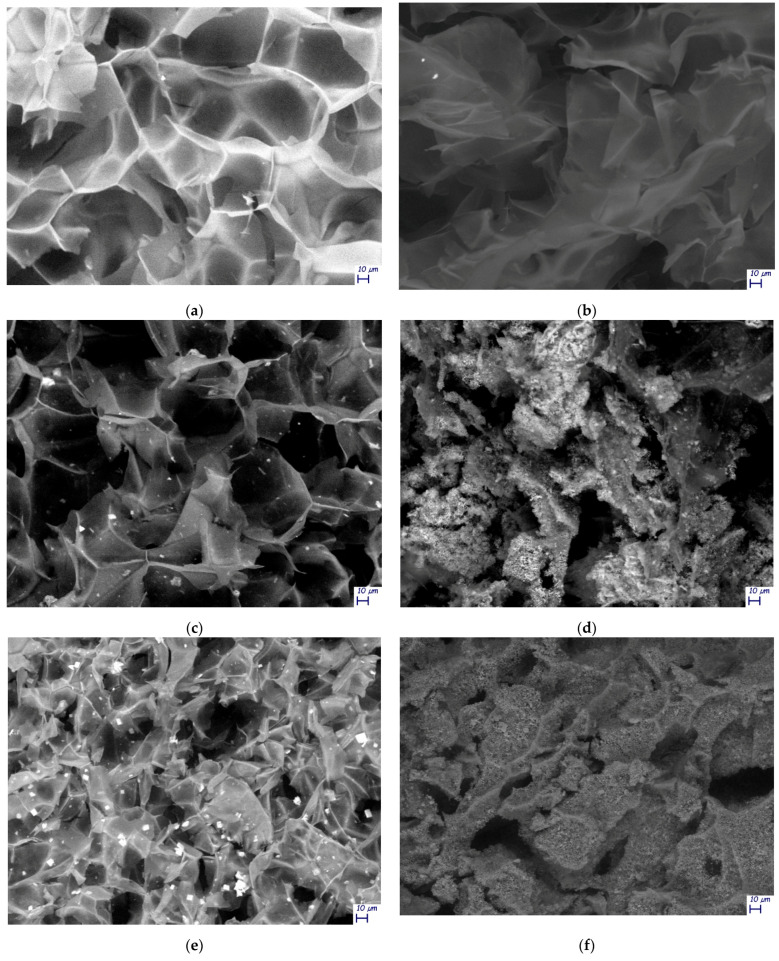
SEM images of: (**a**) TOCNFs before mineralization; (**b**) TOCNFs after mineralization (28 d) and washing step; (**c**) TOCNFs CaP before mineralization; (**d**) TOCNFs CaP after mineralization (28 d) and washing step; (**e**) TOCNFs CaPGO before mineralization; (**f**) TOCNFs CaPGO after mineralization (28 d) and washing step. Scalebar 10 μm. In Figures S6 and S7 are reported SEM images of mineralized TOCNFs CaP and TOCNFs CaPGO acquired at higher magnification.

These results were also confirmed by means of SEM microscopy (Figure 6a–f). In TOCNFs samples, before (Figure 6a) and after (Figure 6b) the mineralization, only the cellular structures typical of lyophilized hydrogels can be observed, without any evidence of formation of HA on its surface. In contrast, in samples TOCNFs CaP (Figure 6c) and TOCNFs CaPGO (Figure 6e) acquired before the mineralization experiments, the HA precursors (CaP and CaPGO) homogeneously dispersed on the cellular structures can be observed. After the mineralization of these samples, the hydrogels structures were covered by spherical HA nanoparticles interlinked with each other (Figure 6d,f), having an average size in the range of 50 nm–150 nm, in good accordance with the literature [41,42].

#### 3.2.3. Cytotoxicity Assay

**Figure 7:**
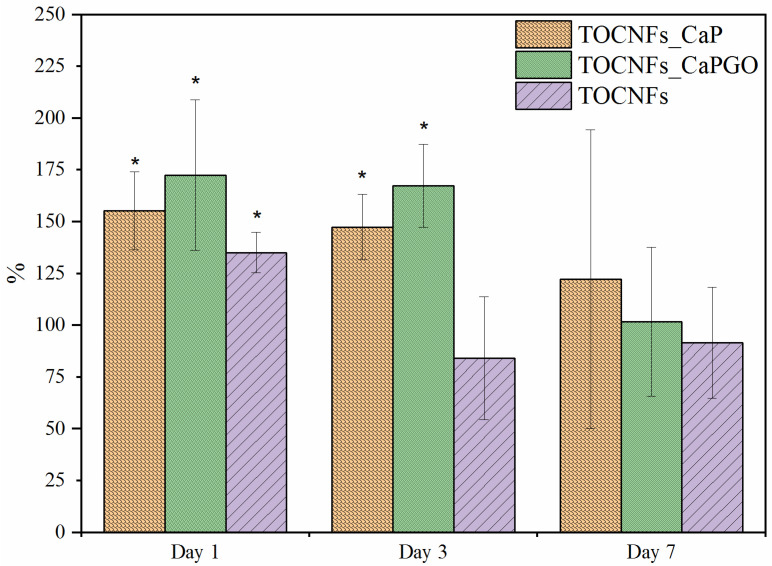
Viability of SAOS-2 cells incubated with eluates obtained from different hydrogels. * Statistically significant differences were evaluated by means of t-student test (*p* < 0.05).

In Figure 7, we reported the metabolic activity of cells cultured in contact with eluates, evaluated by Alamar Blue assay. The viability of cells incubated with material extracts (eluates) was comparable or higher than those of cells cultured under standard conditions. All values are above 70%, and the limit to consider samples non cytotoxic was determined according to the EN ISO 10993-12 standard. These results demonstrated that TOCNFs-based hydrogels do not release any cytotoxic compounds. Moreover, no significant differences were detected between TOCNFs CaP and TOCNFs CaPGO, indicating that, for all time points of incubation, the inorganic components did not affect SAOS-2 cell viability.

## 4. Conclusions

The use of cellulose in tissue engineering is often limited due to the lack of an adhesive site in cells. The possibility of fabricating biocomposites could overcome this drawback. In this work, we started to investigate composite hydrogels that were successfully obtained with both CaP and CaPGO. Their physico-chemical properties can be enhanced through the addition of the inorganic phases within the matrix. These properties can be easily modulated by modifying the amount of inorganic components, depending on the final application. Hydrogels showed a good injectability; indeed, they can be easily extruded in typical conditions employed during surgery practice since relative low loads (1.5–5 N) are needed in order to inject about 2.3 mL of hydrogel in one minute. At the same time, the proposed biocomposites show a good bioactivity; in fact, both CaP and CaPGO hydrogels were able to induce the mineralization process, and an HA layer was observed on composite samples after SBF immersion. This result suggests that the proposed hydrogel can be easily injected in bone defects. Finally, no cytotoxic effect, due to the release of any component from the hydrogel, was shown in any of the samples. However, further investigation on biocompatibility would be necessary to understand potential applications in bone tissue regeneration.

## Figures and Tables

**Table 1:** Specimen’s details.

Sample ID	TOCNFs Dispersion [mg]	HA [mg]	HAGO [mg]	Deionized Water [μL]
TOCNFs_CaP	445	5	-	50
TOCNFs_CaPGO	445	-	5	50
TOCNFs	445	-	-	55

## Data Availability

Data is contained within the article or supplementary material.

## Supplementary Materials

The following are available online at https://www.mdpi.com/article/10.3390/ma14164511/s1, Figure S1: Rheological characterization: determination of LVR. Strain sweep in the range 0.01–100% with constant frequency at 1 Hz, Figure S2: Schematic representation of custom injectability ex-perimental setup, Figure S3: FT-IR of pristine cellulose (black line), oxidized cellulose (red line), TOCNFs (blue line), Figure S4: (a) Nanoparticles aspect, (b) FT-IR of CaP (red line) and CaPGO (green line) nanoparticles, (c) XRD diffractogram of CaP nanoparticles (d) XRD diffractogram of CaPGO nanoparticles, Figure S5: (a) TG-DTA curves of (a) TOCNFs, (b) TOCNFs_CaP, (c) TOC-NFs-CaPGO, Figure S6: SEM images of TOCNFs CaP after mineralization (28 d) and washing step, Figure S7: SEM images of TOCNFs CaPGO after mineralization (28 d) and washing step.
