# Supplementary material for: Bioactive Hydrogels: Design and Characterization of Cellulose-Derived Injectable Composites
Source: Materials (Basel). 2021 Aug 11;14(16):4511. doi: 10.3390/ma14164511 (PMC8398032; doi:10.3390/ma14164511)
Supplement: Supplementary file 1 [file materials-14-04511-s001.zip › materials-1298952-supplementary.pdf]

materials

Supporting information

Bioactive Hydrogels: Design and Characterization of Cellulose-Derived Injectable Composites

Andrea Fiorati [1,2,*], Cristina Linciano [1], Camilla Galante [1], Maria Grazia Raucci [3] and Lina Altomare [1,2]

1 Department of Chemistry, Materials, and Chemical Engineering "G. Natta"—Politecnico di Milano, Piazza Leonardo da Vinci 32, I-20133 Milano, Italy; cristina.linciano@mail.polimi.it (C.L.); camilla.galante@mail.polimi.it (C.G.); lina.altomare@polimi.it (L.A.)
2 INSTM National Interuniversity Consortium of Materials Science and Technology, Politecnico di Milano Local Unit, Piazza Leonardo da Vinci 32, I-20133 Milano, Italy.
3 Institute of Polymers, Composites and Biomaterials (IPCB), National Research Council (CNR) Viale J.F. Kennedy, 54 Mostra d'Oltremare Pad.20, 80125 Naples, Italy; mariagrazia.raucci@cnr.it
* Correspondence: andrea.fiorati@polimi.it

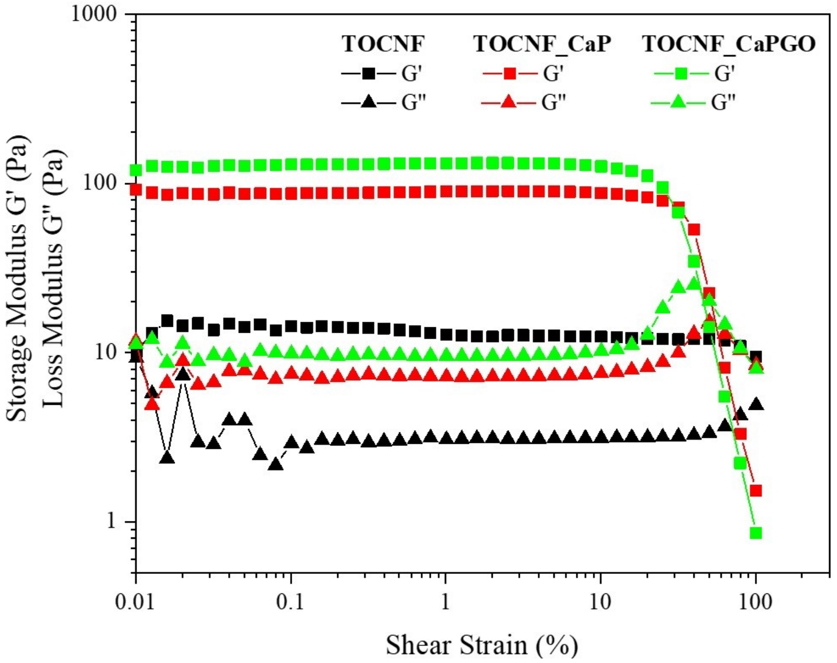

Figure S1. Rheological characterization: determination of LVR. Strain sweep in the range 0.01–100% with constant frequency at 1 Hz.

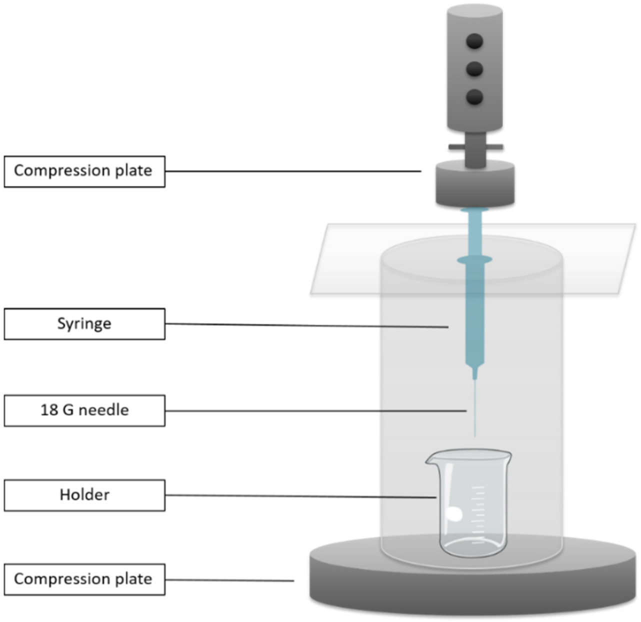

Figure S2. Schematic representation of custom injectability experimental setup.

Figure S3 reports the FT-IR of pristine cellulose, oxidized cellulose and TEMPO oxidized cellulose nanofibers dispersed at pH 7. The occurrence of TEMPO oxidation is demonstrated by the peak at 1731 cm^{-1} which is present only on the TOCNFs spectra. This peak is associated with the C=O stretching vibration of the carboxylic acids and it increases with the oxidation degree of the different TOCNFs [1]. Moreover, the spectra registered the characteristic cellulose peaks: at 1057 cm^{-1} corresponding to the vibration of C–O–C on the pyranose ring skeletal; at 1163 cm^{-1}, related to C–O–C asymmetric bridge stretching of the β-glycosidic linkage; at 1203 cm^{-1} for C–O–H in-plane deformation; on the plateau are recognized three peaks corresponding to H–C–H wagging vibration (1317 cm^{-1}), C–H deformation vibration (1371 cm^{-1}) and H–C–H and O–C–H in-plane deformation (1425 cm^{-1}); at 2904 cm^{-1} there is a peak assigned to C–H stretching. Finally, a peak corresponding to O–H bending at 1615 cm^{-1} and a large and broad absorption at 3200–3700 cm^{-1} related to the O–H stretching vibrations are detected, indicating the level of water absorption.

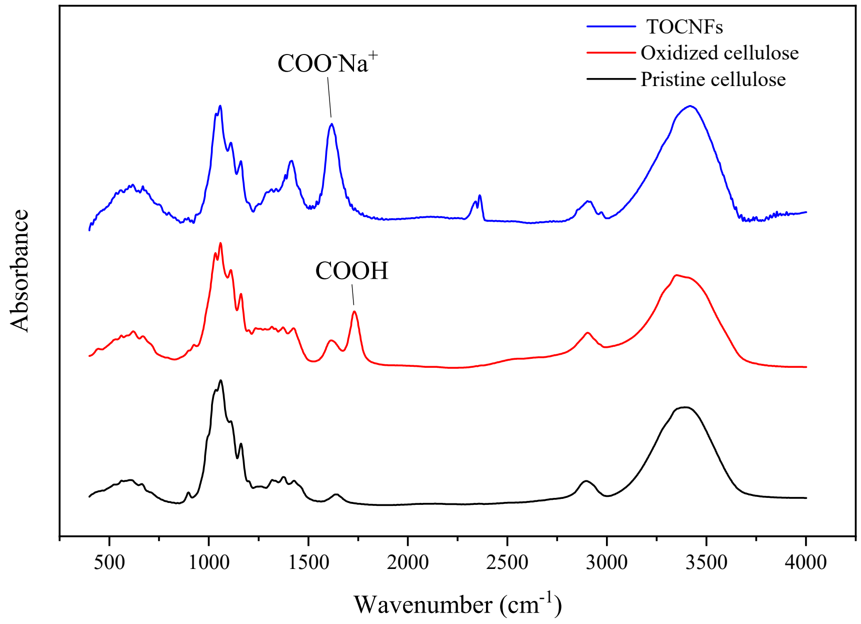

Figure S3. FT-IR of pristine cellulose (black line), oxidized cellulose (red line), TOCNFs (blue line).

In Figure S4 were reported the nanoparticles aspect and the FT-IR and XRD characterization of CaP and CaPGO powders.The CaP FT-IR spectra was attributed, according with literature [2–4], as follow: the bands between 1030–1120 cm^{-1} to the asymmetrical stretching of PO_4^{3-} while the peaks at 565 and 604 cm^{-1} to the bending mode of PO_4^{3-}. The symmetric stretching modes of the phosphate group were responsible of signal at 962 cm^{-1} and the band at 1638 cm^{-1} to the bending mode of O–H residues. The sharp peak at 820 and 1380 cm^{-1} were attributed to nitrate groups (NO^{3-}) resulting from synthesis residuals [5,6].

FT-IR analysis of the CaPGO composite shows no particular differences in composition from CaP. the band corresponding to C-O of the GO can possibly be observed at 1068 cm^{-1} although it can be partly overlapped with the peaks of the phosphate groups, typical of the CaP spectra. Hydroxyl groups are observed at 1639 cm^{-1} (bending mode) as reported previously in CaP spectra, while the typical band of carboxyl groups (1730cm^{-1}) is absent, suggesting the interaction of CaP nanoparticles with functionalized surface groups of GO as stated by Lopes and co-worked [7].

Powder X-ray diffraction (XRD) patterns were reported in the 2θ range 0–60°. The crystalline phases were identified from a comparison of the registered patterns with the data provided by Profex software [8]. Analyzing the diffraction peaks of CaP, it is possible to identify several phases belonging to different calcium phosphates. In particular calcium hydrogen phosphate dihydrate ($CaHPO_4{\cdot}2H_2O$), a precursor of hydroxyapatite, is detected at 11.7° with a strong and sharp peak, indicating a high crystallinity [6]. The latter, also known as brushite (marked with "#" in Figure S2), is also visible at 2θ values of 17.9°, 20.9°, 23.5°, 34.1° and 37.1°. The peaks at 13.1°, 26.5°, 30.2°, 30.5°, 31.1°, 32.6°, 36.1°, 40.1° and 49.3° match with calcium hydrogen phosphate, commonly called monetite ($CaHPO_4$, marked with "@" in Figure S2). Whereas, 2θ values of 28.9°, 32.9°, 35.5°, 39.1°, 39.9°, 49.5°, 53.2° were indexed to be hydroxyapatite (HA) (marked with "*" in Figure S2), although the observed peaks tended to be less intense than those reported in the literature [5,9]. Moreover, some characteristic peaks of stoichiometric HA, usually present at 31.8°, 32.2° and 46.7°, were not observed [5,9]. In general, HA samples demonstrated a characteristic diffraction pattern of low crystalline and/or nanometric hydroxyapatite and monetite phases, as indicated by the broad and poorly defined peaks [7].

The diffractogram of CaPGO also shows the presence of several phases (Figure S2 d). The typical peaks of brushite are not detected while intermediate phases are visible. These may be formed during the dehydration process of brushite that occurs as a result of freeze-drying of the sample. The consequence is a predominance of monetite phase, already observed in the XRD analysis of HA, and crystalline calcium pyrophosphate ($Ca_2P_2O_7$, CPP, marked with "°" in Figure S2) [10]. From the literature the GO exhibits a strong and sharp peak located at 2θ 11.4°, anyway this characteristic signal is not observed in the HAGO composite, which may be due to the lower GO content in the composite [9].

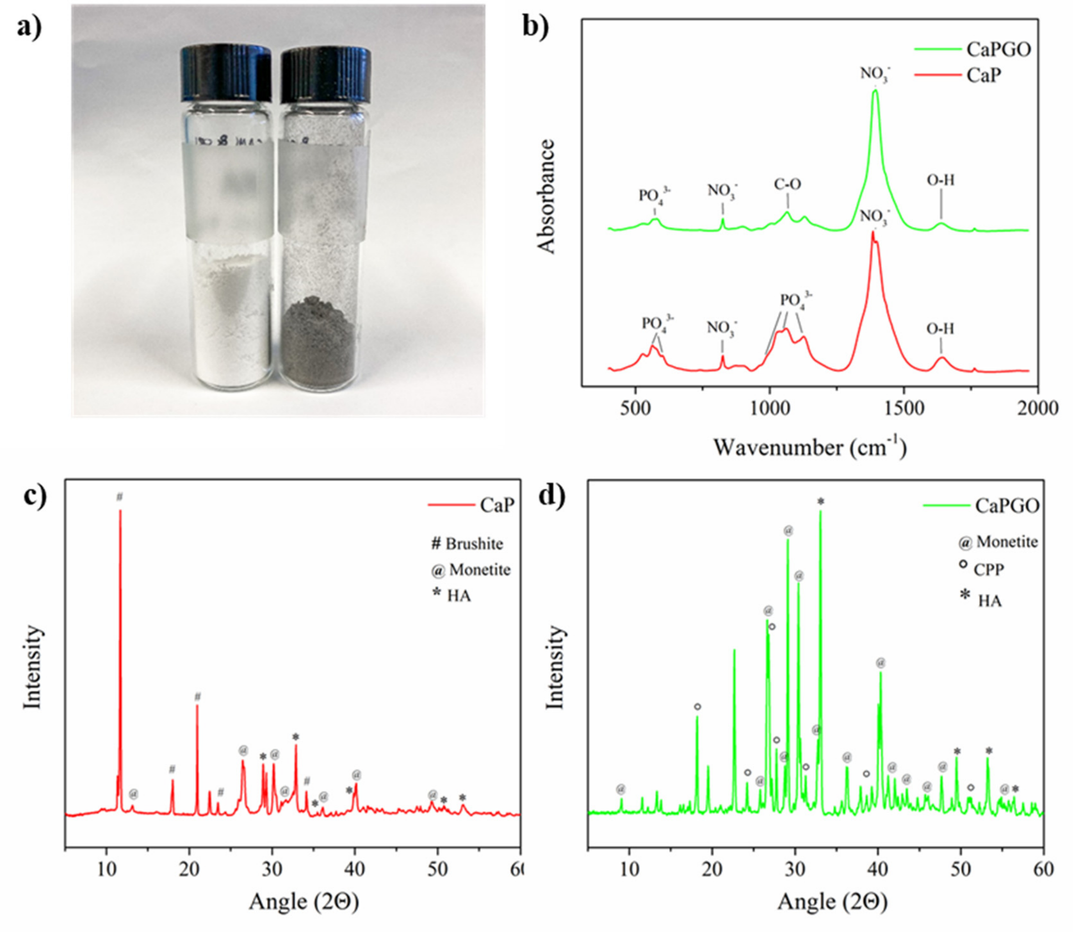

Figure S4 (**a**) Nanoparticles aspect, (**b**) FT-IR of CaP (red line) and CaPGO (green line) nanoparticles, (**c**) XRD diffractogram of CaP nanoparticles (**d**) XRD diffractogram of CaPGO nanoparticles.

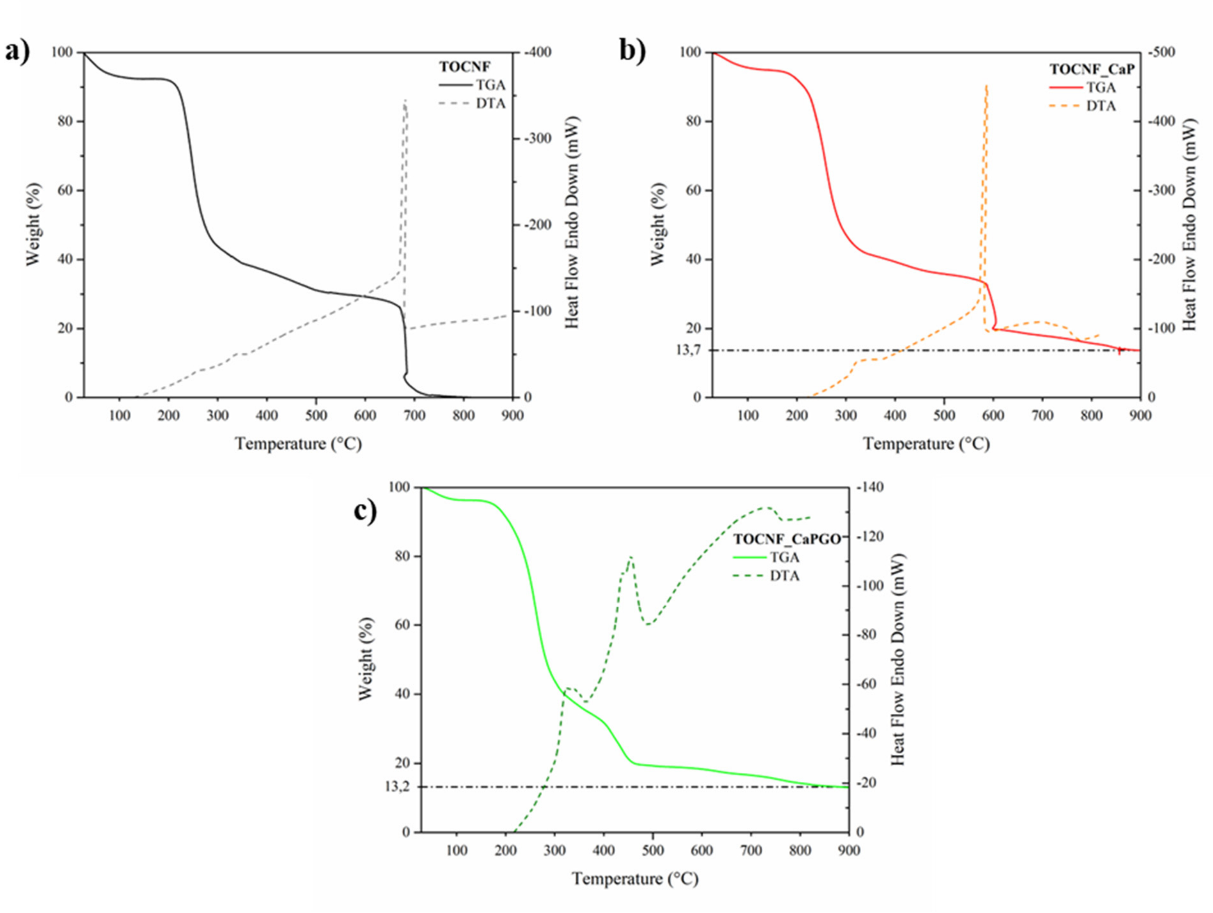

Figure S5. (**a**) TG-DTA curves of (**a**) TOCNFs, (**b**) TOCNFs_CaP, (**c**) TOCNFs-CaPGO.

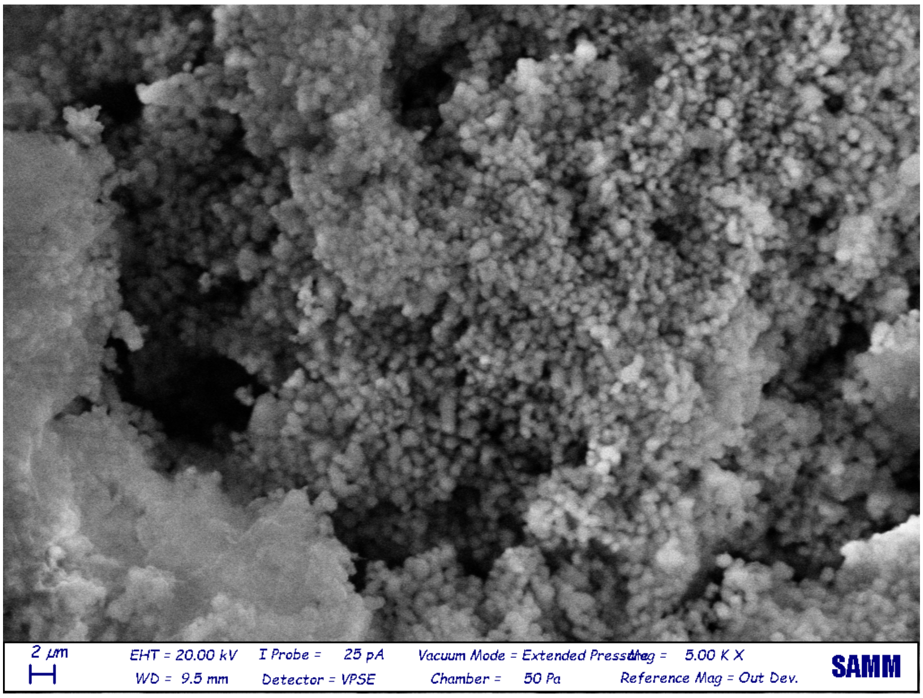

Figure S6. SEM images of TOCNFs_CaP after mineralization (28 d) and washing step.

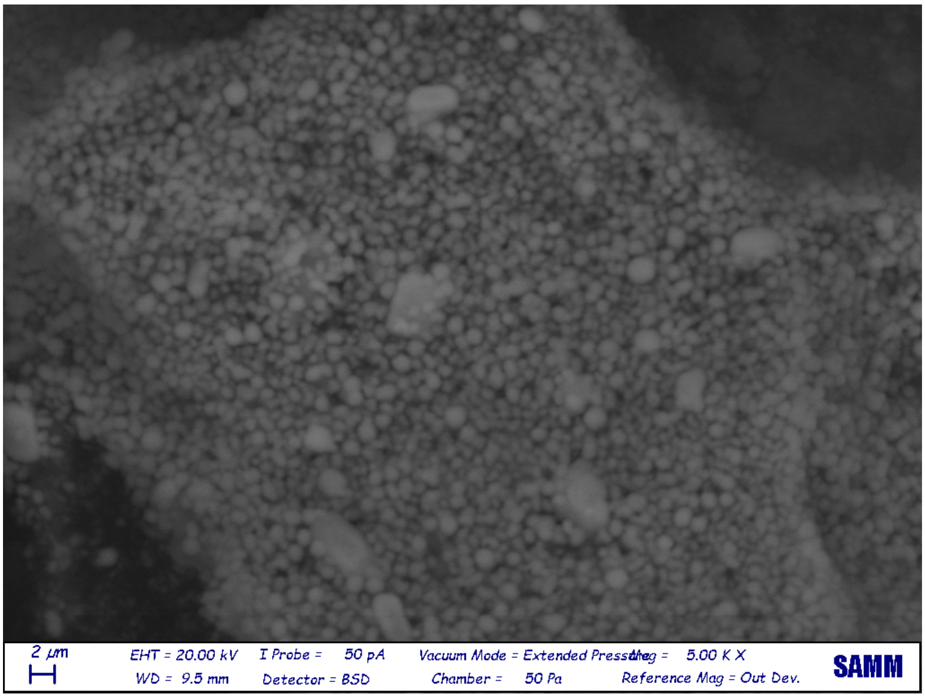

Figure S7. SEM images of TOCNFs_CaPGO after mineralization (28 d) and washing step.

References

1. Melone, L.; Bonafede, S.; Tushi, D.; Punta, C.; Cametti, M. Dip in colorimetric fluoride sensing by a chemically engineered polymeric cellulose/bPEI conjugate in the solid state. *RSC Adv.* **2015**, *5*, 83197–83205.
2. Berzina-Cimdina, L.; Borodajenko, N. Research of Calcium Phosphates Using Fourier Transform Infrared Spectroscopy. In *Infrared Spectroscopy-Materials Science, Engineering and Technology*; IntechOpen Limited: London, 2012.
3. Rhee, S.-H.; Tanaka, J. Self-assembly phenomenon of hydroxyapatite nanocrystals on chondroitin sulfate. *J. Mater. Sci. Mater. Med.* **2002**, *13*, 597-600.
4. Wang, L.; Li, C. Preparation and physicochemical properties of a novel hydroxyapatite/chitosan–silk fibroin composite. *Carbohydr. Polym.* **2007**, *68*, 740-745.
5. Destainville, A.; Champion, E.; Bernache-Assollant, D.; Laborde, E. Synthesis, characterization and thermal behavior of apatitic tricalcium phosphate. *Mater. Chem. Phys.* **2003**, *80*, 269-277.
6. Raucci, M.G.; Giugliano, D.; Longo, A.; Zeppetelli, S.; Carotenuto, G.; Ambrosio, L. Comparative facile methods for preparing graphene oxide-hydroxyapatite for bone tissue engineering. *J. Tissue Eng. Regen. Med.* **2017**, *11*. doi: 10.1002/term.2119.
7. Lopes, C.C.; Pinheiro, W.A.; Navarro da Rocha, D.; Neves, J.G.; Correr, A.B.; Ferreira, J.R.M.; Barbosa, R.M.; Soares, J.R.F.; Santos, J.L.; Prado da Silva, M.H. Nanocomposite powders of hydroxyapatite-graphene oxide for biological applications. *Ceram. Int.* **2021**, *47*. doi: 10.1016/j.ceramint.2020.11.107
8. Doebelin, N.; Kleeberg, R. *Profex* : a graphical user interface for the Rietveld refinement program *BGMN*. *J. Appl. Crystallogr.* **2015**, *48*. doi: 10.1107/S1600576715014685
9. Gong, M.; Zhao, Q.; Dai, L.; Li, Y.; Jiang, T. Fabrication and Friction Coefficient of Graphene Oxide Reinforced Hydroxyapatite Composite. *J. Nanosci. Nanotechnol.* **2018**, *18*. doi: 10.1166/jnn.2018.14253
10. Mouatamid El Hazzat; Adnane El Hamidi; Mohammed Halim Complex phase evolution of Brushite-based calcium phosphate $CaHPO_4 \cdot 2H_2O$ using in situ high temperature X-ray diffraction and thermal analysis. *Research square* **2020**. doi: 10.21203/rs.3.rs-44209/v1
